# *EpiCas-DL*: Predicting sgRNA activity for CRISPR-mediated epigenome editing by deep learning

**DOI:** 10.1016/j.csbj.2022.11.034

**Published:** 2022-11-19

**Authors:** Qianqian Yang, Leilei Wu, Juan Meng, Lei Ma, Erwei Zuo, Yidi Sun

**Affiliations:** a Institute of Neuroscience, CAS Center for Excellence in Brain Science and Intelligence Technology, Chinese Academy of Sciences, Shanghai, China; b Zhengzhou Research Base, State Key Laboratory of Cotton Biology, School of Agricultural Sciences, Zhengzhou University, Zhengzhou 450001, Henan, China; c Shenzhen Branch, Guangdong Laboratory for Lingnan Modern Agriculture, Genome Analysis Laboratory of the Ministry of Agriculture, Agricultural Genomics Institute at Shenzhen, Chinese Academy of Agricultural Sciences, Shenzhen, China

**Keywords:** CRISPR-mediated epigenome editing, Deep learning, Convolutional neural network, *EpiCas-DL*, Gene silencing, Gene activation

## Abstract

CRISPR-mediated epigenome editing enables gene expression regulation without changing the underlying DNA sequence, and thus has vast potential for basic research and gene therapy. Effective selection of a single guide RNA (sgRNA) with high on-target efficiency and specificity would facilitate the application of epigenome editing tools. Here we performed an extensive analysis of CRISPR-mediated epigenome editing tools on thousands of experimentally examined on-target sites and established *EpiCas-DL*, a deep learning framework to optimize sgRNA design for gene silencing or activation. *EpiCas-DL* achieves high accuracy in sgRNA activity prediction for targeted gene silencing or activation and outperforms other available *in silico* methods. In addition, *EpiCas-DL* also identifies both epigenetic and sequence features that affect sgRNA efficacy in gene silencing and activation, facilitating the application of epigenome editing for research and therapy. *EpiCas-DL* is available at http://www.sunlab.fun:3838/EpiCas-DL.

## Graphical abstract

**Figure:**
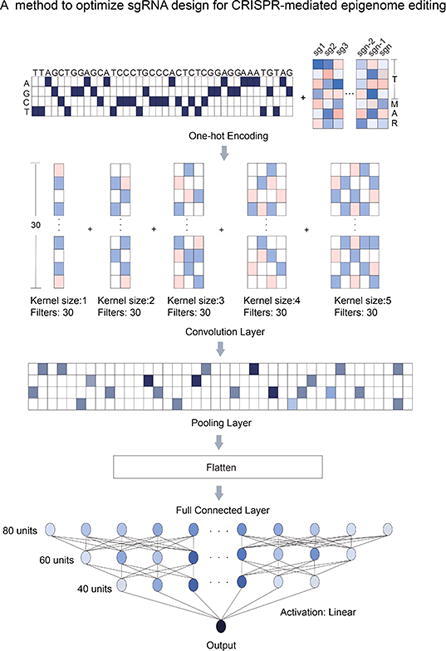

## 1 Introduction

The CRISPR systems have emerged as powerful DNA editing tools to flexibly target genomic DNA in various species and cell types. CRISPR-associated (Cas) nucleoproteins could achieve genomic specificity with the assistance of target-specific single-guide RNA (sgRNA) [1]. Most Cas proteins generate knock-outs or knock-ins by exploiting cellular DNA repair pathways after introducing DNA breaks at targeted loci [1]. Base editors and prime editors were consecutively engineered to enable precise editing for the correction or installation of mutations related to pathogenic diseases [2], [3], [4]. Additionally, the fusion of catalytically inactive dead Cas9 (dCas9) with gene-regulatory proteins has generated CRISPR interference and activation tools (CRISPRi/a) for transcriptional down-regulation and up-regulation [5], [6], [7], [8], [9]. Programmable epigenome editing tools could be used to manipulate gene expression without altering the underlying DNA sequences [10], [11]. The recently developed CRISPRoff could effectively produce transcriptional regulation by establishing DNA methylation and repressive histone modifications and achieve long-term gene silencing [12]. These epigenome gene-editing tools could be used to systematically regulate gene expression for pathogenetic genes or high-throughput genetic screens.

The selection of sgRNAs with high on-target efficiency and specificity is critical for the application of CRISPR editing systems [13]. So far, predictive algorithms have been developed to select sgRNAs with improved editing efficiency [14], [15], [16]. Although many of these algorithms showed good predictability in their training datasets, the generalization performances of these models are limited [17]. Most of these tools only rely on the sequence characteristics of the sgRNA and the target sequence, but factors beyond genomic context also play important roles in impacting the sgRNA activity of CRISPR editing. Previous studies have shown nucleosome occupancy could impede the binding of Cas9 to DNA, and gene expression level and chromatin accessibility of the target site also have the potential to influence Cas9 activity [14], [18], [19]. These features provide additional dimensions for the editing outcomes of CRISPR systems, particularly important for applications of epigenome editing tools, which require the recruitment of DNA methyltransferases or histone modification proteins [20]. Therefore, we hypothesized that building a prediction algorithm that incorporates all these epigenetic features could greatly facilitate the optimal design of sgRNAs in epigenome editing systems.

Here, we present a comprehensive deep learning framework to predict the sgRNA on-target editing efficiency of epigenome editing tools. Our approach, named **Epi**genome **C**RISPR-**as**sociated (**Cas**) **D**eep **L**earning (*EpiCas-DL*), is based on a deep neural network and takes epigenetic information from different cell types into consideration for model training and prediction. The resulting models achieved high predictive efficacy on data from CRISPRoff, CRISPRi, and CRISPRa screening datasets (Table S1). Our *EpiCas-DL* model competes favorably with the available state-of-the-art tools in independent validation cohorts and different cell types. Further, the model automates the identification of sequence and epigenetic features and learns which features are important for optimized on-target editing, which helps to decipher the mechanism of CRISPR-based epigenome editing in a much more efficient way. The *EpiCas-DL* can be easily accessed at https://www.sunlab.fun:3838/EpiCas-DL.

## 2 Methods

### 2.1 Data source

We collected a total of nine independent datasets using three epigenome editing tools for the model development and comparison in this study (Table S1). 1) Three sgRNA datasets with CRISPRoff editing outcomes, CRISPRoff_tiling, CRISPRoff_genome, and four endogenous genes datasets, were collected from James K. Nunez et al [21]. CRISPRoff_tiling dataset contains 111,682 sgRNAs within ± 2.5 kb from the TSS of 520 genes (Table S1). CRISPRoff_genome dataset contains 20,221 sgRNAs across 18,779 genes. The four endogenous gene dataset contains 326 sgRNAs for H2B gene, 415 sgRNAs for CLTA gene, 392 sgRNAs for RAB11A gene, and 528 sgRNAs for VIM gene. 2) Four datasets were included with CRISPRi editing. CRISPRi_activityscore [20] dataset containing 18,079 sgRNAs on 1539 genes, hCRISPRi-v2 [20] containing 199,523 sgRNAs on 18,549 genes, CRISPRi_genome [21] containing 107,595 sgRNAs on 14,361 genes, and CRISPRi_K562 [12] containing 111,283 sgRNAs on 520 genes. CRISPRi_activityscore and hCRISPRi-v2 were collected from Horlbeck et al [20]. CRISPRi_genome and CRISPRi_K562 were carefully curated from published literature [6], [12], respectively. 3) The hCRISPRa-v2 and CRISPRa_activityscore datasets with CRISPRa editing were included [12]. The hCRISPRa-v2 contains 198,756 sgRNAs on 18,495 genes and CRISPRa_activescore contains 2779 sgRNAs on 236 genes. Detailed information for each dataset is shown in Tables S1 and S3.

### 2.2 Data preprocessing

The efficiency of sgRNAs in CRISPRoff and CRISPRi datasets in addition to CRISPRi_activityscore and CRISPRa_activityscore were originally represented by the phenotype score, where smaller scores indicate a stronger growth defect. To normalize the phenotype scores from different datasets, we firstly transformed the negative scores of sgRNAs from gene silencing studies into positive ones to make sure large values represent higher efficiency. Then we calculated activity scores for the growth-based screens by normalizing the phenotype score of each sgRNA with the absolute average score of the top 3 sgRNAs for each gene (Fig. S2a). The CRISPRoff_genome dataset only included one or two sgRNAs for each gene and was not applicable to gene-level normalization, so we used the transformed phenotype values as activity scores. The activity scores for each dataset were used as input for the following analysis. In the classification model, we assigned the sgRNAs into two categories, sgRNAs with high gene silencing or activation activities were labeled as “1”, and the others were labeled as “0”. The cutoff of label categorization for each dataset was defined according to the criteria reported in the respective experiment [7], [12], [20]. In particular, the phenotype scores lower than −0.1 were considered as highly active and labeled as “1”, and activity scores greater than 0.75 were considered as “1” for CRISPRi_activityscore and CRISPRa_activityscore datasets where no phenotype scores were provided.

For the epigenetic features, we retrieved the TSS position information from the FANTOM consortium [12], [22], which showed a significant improvement over the definition for TSS in Ensemble/GENCODE [23]. We next calculated both the distance to primary Cap Analysis of Gene Expression AND deep Sequencing (CAGE) TSS and secondary CAGE TSS, and thus get four values representing nucleosome positions for each sgRNA. The chromatin accessibility for each sgRNA was estimated using genome-wide ATAC-seq data collected from the chromatin accessibility database ATACdb [24]. The RNA-seq and whole-genome bisulfite sequencing (WGBS) data were retrieved from Gene Expression Omnibus (GEO) database to quantify gene expression and DNA methylation level for each sgRNA, respectively (Table S2). Each sgRNA was assigned for the four types of epigenetic features based on chromosome position using custom scripts written in Python 3.6 with “pybedtools” (v0.9.0) on the CentOS system. All the epigenetic features were min–max normalized before model input.

### 2.3 Encoding for sequence and epigenetic features

**Fig. 1:**
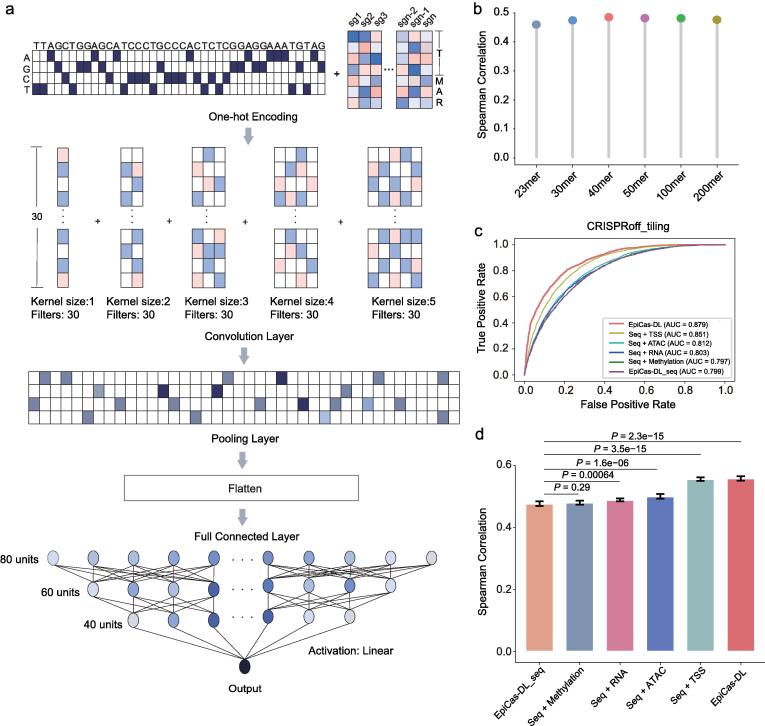
Implementation details of *EpiCas-DL* on the CRISPRoff dataset. (a) Overview of EpiCas-DL model. For a specific DNA sequence, the 40 bases pair (bp) nucleotide sequence is one-hot encoded and represented by four channels as A-, G-, C-, or T-channel, and each epigenetic feature is considered as one channel with continuous values. The “T”, “A”, “M” and “R” were abbreviated for “nucleosome positioning estimated by distance to TSS”, “chromatin accessibility revealed by ATAC-seq”, “DNA methylation”, and “RNA expression”, respectively. (b) Evaluation of *EpiCas-DL* performance on models with different lengths of input sequence using a regression model. Spearman correlation was calculated by predicted activity scores and observed ones in the CRISPRoff dataset. (c)-(d) Performance comparison of *EpiCas-DL* models with different epigenetic features in the CRISPRoff dataset using classification schema (c) and regression schema (d). “seq” represents sequence. Error bars show the s.e.m. from 10-folds cross-validation. *P* values were calculated by Student’s *t*-test.

One-hot encoding strategy was used to formulate the sgRNA sequence into an image-like coding scheme (Fig. 1a). We retrieved the 9 bp upstream and 8 bp downstream sequences together with the 23 bp target sequence for each sgRNA, and the 40 bp DNA sequence was transformed into a matrix of four rows and 40 columns. Each row represents a base channel with “0″ and “1” to indicate the presence or absence of a specific base A, T, G, or C. The normalized epigenetic features were converted to the same data format as the sequence matrix but with continuous values. The final input data for each sgRNA was an 11 × 40 matrix, including 4 sequence features, 4 nucleosome positioning features, 1 chromatin accessibility feature, 1 DNA methylation feature, and 1 gene expression feature.

**Fig. 2:**
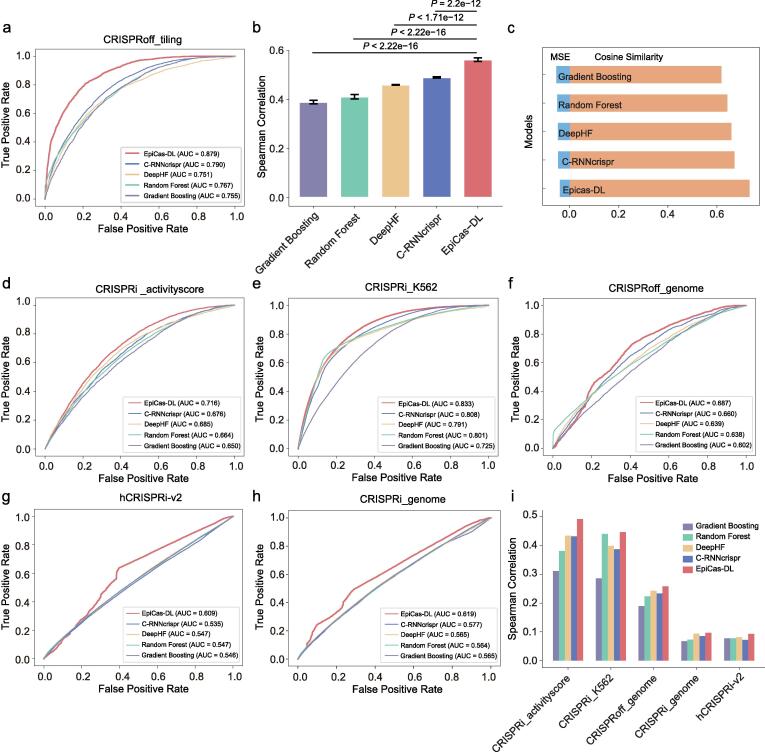
Evaluation of *EpiCas-DL* prediction performance in different scenarios. (a) The comparison of *EpiCas-DL* with other models using classification schema. (b) The performance comparison between *EpiCas-DL* and other models using a regression model. Error bars show the s.e.m. from 10-folds cross-validation. *P* values were calculated by Student’s *t*-test. (c) Comparison of different models using MSE (mean squared error) and cosine similarity in the CRISPRoff_tiling dataset. (d)-(h) Performance comparison of different models on the sgRNA activity score prediction in different gene silencing datasets using classification schema. (i) Performance comparison of different models on the sgRNA activity score prediction in different gene silencing datasets in the regression model.

### 2.4 Model development

In the development of *EpiCas-DL*, we randomly divided this dataset into training and testing datasets by 9:1 and performed 5-folds cross-validation in the training dataset for hyperparameter optimization. *EpiCas-DL* applies a convolutional neural network (CNN) to learn the underlying features from the input sequence and epigenome information for sgRNA activity prediction. The convolution layer contains five different sizes of filters (30 1-nt filters, 30 2-nt filters, 30 3-nt filters, 30 4-nt filters, and 30 5-nt filters). MaxPooling was adopted in our pooling layer, followed by three fully connected layers with 80 units, 60 units, and 40 units. To avoid overfitting during model training, we added the dropout function with the value of 0.4 after batch normalization. The rectified linear unit (Relu) activation was used in the convolution layer and fully connected layer, and linear activation was used in the output layer. To help select the hyperparameters in our CNN model, we applied a Bayesian optimization package, GPyOpt, which has been demonstrated to be more reliable than random grid search [25]. We set the initial random searching points as 30, and 300 acquisitions were executed in an attempt to obtain the global optimal value during the iteration.

### 2.5 Model comparison

To compare the performance of *EpiCas-DL* with other four algorithms (*Gradient Boosting*, *Random Forest*, *DeepHF*, and *C-RNNcrispr*), we applied the default configurations of each method to build models using the same input as *EpiCas-DL* containing both sequence and epigenetic features. We saved the model with the highest performance on the validation set during training, and made predictions on the test set using the trained model and calculated the spearman correlation coefficients and ROC-AUC for regression and classification models, respectively. Scikit-learn 0.24.2 and Tensorflow 2.4.1 were used as the backend for machine learning algorithms and deep learning models, respectively.

### 2.6 Feature identification

Feature identification was achieved using a recently developed method, Tree SHAP (Shapley Additive exPlanations), which associated the SHAP values with the XGBoost algorithm [26]. The sequence and epigenetic features were extracted by the in-house python script and fitted into XGBoost. Mean |SHAP| values, the average impact on model output magnitude, were interpreted on the training dataset by TreeExplainer. The top 30 features with the highest |SHAP| values were presented, and all the values were listed in Table S4.

To better demonstrate the specific sequence features predictable for the editing efficiency of epigenome editing systems, we further identified features contributing to the performance of *EpiCas-DL* by DeepSHAP [27]. DeepExplainer function was applied to calculate SHAP values from the CRISPRoff and CRISPRa models, and SHAP values for randomly selected 1000 sgRNAs were averaged and presented by a histogram.

## 3 Results

### 3.1 The development of *EpiCas-DL* for sgRNA efficacy prediction for CRISPR-mediated epigenome editing

To explore the relationship between epigenome editing outcomes and epigenetic features, we collected data from 8 gene silencing or activation screening datasets available and calculated the editing efficiency represented by activity scores for a total of 769,918 sgRNAs with experimentally examined knock-down or knock-up efficiencies on 18,975 genes (Fig. S1a and Table S1). The gene silencing datasets included 2 CRISPRoff datasets and 4 CRISPRi datasets, and gene activation datasets included 2 genome-wide CRISPRa datasets from HEK293T and K562 cells, the two most widely used cell lines in biological studies. The epigenetic features including DNA methylation, RNA expression, chromatin accessibility, and nucleosome positioning for HEK293T cells and K562 cells were retrieved from Gene Expression Omnibus (GEO), chromatin accessibility database (ATACdb), and FANTOM consortium annotations [22], [24], [28], [29], [30], [31], [32], respectively (Table S2). Low correlations were observed between each pair of these epigenetic features (Fig. S1b), suggesting their independent roles in affecting the editing efficiency of epigenome editing tools. We firstly compared the editing efficiency of sgRNA targeting sites with or without DNA methylation and found that sites with DNA methylation showed higher gene repression efficiency in the two CRISPRoff datasets (Fig. S2a), suggesting that endogenous DNA methylation could improve the silencing effects of targeted genes of CRISPRoff. Besides, highly expressed genes also showed higher gene repression or activation efficiency than lowly expressed genes in both the gene silencing and activation datasets (Fig. S2b). Similarly, the target sites with high chromatin accessibility also revealed higher editing efficiency than those with low accessibility in all the examined datasets (Fig. S2c). Considering the influence of chromatin structure on gene activity, these results were in line with previous studies showing that chromatin structure may influence the efficacy of CRISPR-mediated genome editing [33], [34], [35]. We also evaluated the relationship of sgRNA activity with nucleosome positioning quantified by the distance between the target site and the nearest transcription start site (TSS) and observed that sgRNAs targeting nucleosome-deprived regions adjacent to the TSS showed the highest repression efficiency (Fig. S2d–f), consistent with previous studies suggesting the impediment of nucleotide occupancy to dCas binding to DNA [36], [37].

We next attempted to develop computational models that predict the outcomes of epigenome editing systems. Previous studies have used machine learning algorithms to predict the activities of CRISPRi or CRISPRa [19], [20], but deep learning-based methods often outperformed conventional machine learning-based models in predicting efficiencies or outcomes of CRISPR nucleases [14], [19], [38], [39]. Using deep learning frameworks and the CRISPRoff_tiling library [12] as the training dataset, we firstly generated a convolutional neural network (CNN), named *EpiCas-DL*, to predict the gene repression efficiency of CRISPRoff (Fig. 1a). The *EpiCas-DL* algorithm includes one convolutional layer, one Maxpooling layer, and three fully connected layers, with the sgRNA sequences and epigenetic features as input (Fig. 1a). The sgRNA sequences were encoded with one-hot feature representation, and the epigenetic features of each target site were put into the model with min–max normalized continuous values (see Methods; Fig. 1a). Besides, a total of 150 filters with different sizes were used parallelly in the convolutional layer (30 1-nt filters, 30 2-nt filters, 30 3-nt filters, 30 4-nt filters, and 30 5-nt filters), and the rectified linear unit (ReLU) activation function was used to transform the summed weighted input of the convolutional layer. Then the outputs of the convolutional layers were concatenated in the pooling layer and flattened as inputs for the fully connected layers. Here, 80 units, 60 units, and 40 units were selected for three fully connected layers as the best hyperparameters using GPyOpt [25]. We also added drop-out functions after the pooling and fully-connected layers to avoid overfitting. To achieve rigorous evaluations of *EpiCas-DL*, both classification and regression models were built to enable a comprehensive comparison for the efficiency prediction. We randomly divided the dataset into a training dataset (90%) and a testing dataset (10%) and calculated the area under the receiver operating characteristic (ROC) curve (AUC) or Spearman correlation coefficient between the true and predicted values on the testing dataset by 10-fold cross-validations.

The efficiency of CRISPR-Cas systems has been reported to be associated with the context surrounding the sgRNA binding site [40]. Therefore, we firstly compared the effect of the length of input sequences on the prediction accuracies of *EpiCas-DL* (Fig. 1b). The input of the 23mer model included the 20 base-pair (bp) sgRNA sequences with an NGG protospacer adjacent motif (PAM), and 30mer, 40mer, 50mer, 100mer, and 200mer models contained upstream and downstream sequences in addition to the 23 bp target sequences as input (Fig. S3a). By comparing the Spearman correlation coefficients between the predicted and observed editing efficiencies in the test set, we finally selected the 40mer model for the following analyses (Fig. 1b), which includes a 20 bp protospacer, a 3 bp PAM, a 9 bp upstream and 8 bp downstream sequences of the target site (Fig. S3a). To explore the importance of epigenetic features on the predictive ability of the model, we built 6 classification and 6 regression models by combining one or all of the epigenetic features with the sequence features (Fig. 1c and d). The results showed that the model integrating all the sequence and epigenetic features (*EpiCas-DL*) achieved the best performance in both the classification and regression scheme than models containing only sequence features (*EpiCas-DL_seq*), sequence and methylation (*Seq + Methylation*), sequence and RNA expression (*Seq + RNA*), sequence and chromatin accessibility (*Seq + ATAC*), or sequence and nucleosome positioning features (*Seq + TSS*; Fig. 1c and d). Besides, the loss function during training and validation both converged fast (Fig. S3b), indicating the robustness of our model.

### 3.2 Comparison of *EpiCas-DL* with state-of-art methods

To evaluate the ability of *EpiCas-DL* for predicting the sgRNA activity in gene repression of epigenome editing tools, we firstly compared our *EpiCas-DL* models with four state-of-the-art methods including *Random Forest* [41], *Gradient Boosting* [42], *DeepHF* [15], and *C-RNNcrispr* [43]. Our reasons for choosing these models are as follows: (1) *Random Forest* and *Gradient Boosting* are two powerful machine learning algorithms that were widely used in data prediction. (2) *DeepHF* is based on a recurrent neural network (RNN) and uses an embedding encoding method to encode nucleotide sequences as continuous variables instead of discrete variables. (3) *C-RNNcrispr* applies CNN for feature extraction and includes the bidirectional gate recurrent unit network (BGRU) module to model sequential dependencies of sgRNA sequences in both forward and backward directions. (4) *DeepHF* and *C-RNNcrispr* models also included more features in addition to the sequence features, *DeepHF* considered 21mer sequences and secondary structure features as input, and the inputs of the *C-RNNcrispr* model included 23mer sequences and histone modification features [15].

We first compared the performance of *EpiCas-DL* with the other algorithms using the CRISPRoff dataset and found that the AUC of *EpiCas-DL* achieved 0.879, which was much higher than the other models (*C-RNNcrispr*: 0.790, *DeepHF*: 0.751, *Random Forest*: 0.767*, Gradient Boosting*: 0.755; Fig. 2a). Simultaneously, the regression model of *EpiCas-DL* also significantly outperformed the other models evaluated by the Spearman correlation coefficients between the predicted efficiencies and observed ones (Fig. 2b). In addition, we evaluated the performance of *EpiCas-DL* by two other indexes, mean squared error (MSE) and cosine similarity, and the *EpiCas-DL* model also showed the best performance among the evaluated models (Fig. 2c). An additional issue affecting the model performance is the training time, which is closely related to the algorithm structure. We compared the running time of three deep learning models (*EpiCas-DL*, *DeepHF*, and *C-RNNcrispr*), and found that *EpiCas-DL* showed a significantly shorter training time per epoch than the other models (Fig. S3c), suggesting that *EpiCas-DL* has a refined structure that is suitable for large datasets.

**Fig. 3:**
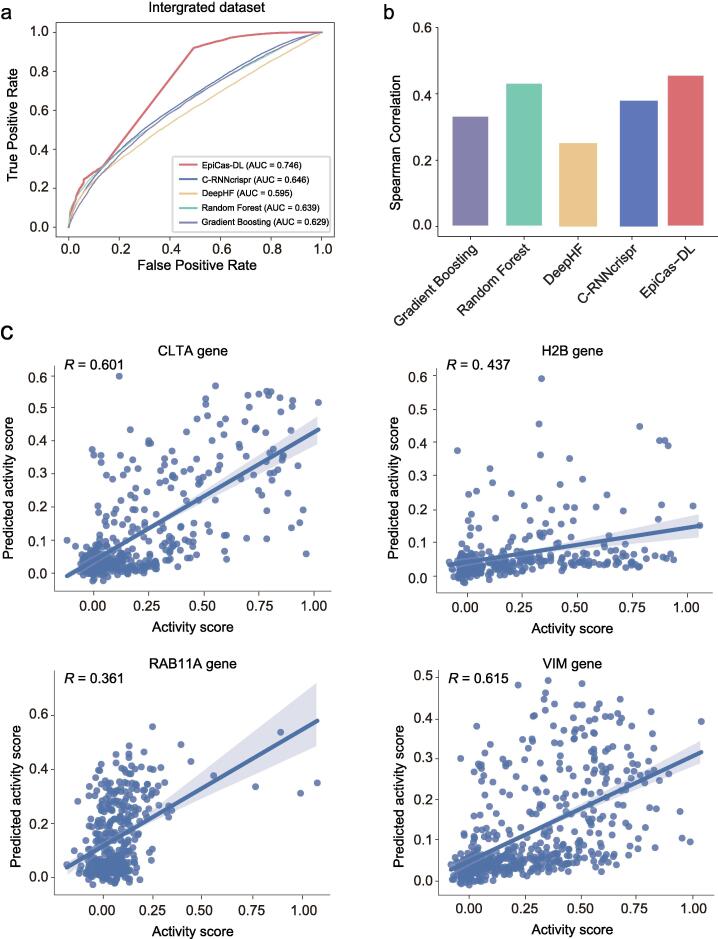
The performance of *EpiCas-DL* in integrated datasets across different cell lines. (a)-(b) Performance comparison of different models on sgRNA efficiency prediction in the integrated test datasets using classification schema (a) or regression schema (b). (c) The correlation between activity scores and predicted ones using *EpiCas-DL* for sgRNAs on the four indicated genes. The correlation coefficients were calculated by the Spearman correlation coefficient.

We next evaluated the prediction ability of *EpiCas-DL* in five independent genome-wide screening datasets, including CRISPRi_activityscore, CRISPRi_K562, CRISPRoff_genome, and hCRISPRi-v2 from K562 cell line, and CRISPRi_genome from HEK293T cell line. For each of the five tested datasets, the *EpiCas-DL* classification model showed the highest ROC-AUCs compared with the other models and outperformed the second-best model (Fig. 2d–h). Similarly, the *EpiCas-DL* regression model also demonstrated much higher prediction accuracies than the other four models in all the tested datasets (Fig. 2i). To further test the generality of our *EpiCas-DL* model across different cell lines, we combined 90 % of the five independent datasets for model training and examined the model performance on the remaining 10 % of the data (see Methods). The results showed that *EpiCas-DL* outperformed the other models with an overall ROC-AUC of 0.746 (Fig. 3a). Moreover, the regression model of *EpiCas-DL* also showed a higher Spearman correlation coefficient than those of other models (Fig. 3b). To visualize the *EpiCas-DL* prediction results intuitively, we additionally tested the performance of *EpiCas-DL* in an independent CRISPRoff_tiling dataset containing 1662 sgRNAs targeting four genes [12]. The predicted efficiencies represented by activity scores showed high consistency with the observed ones for all the tested genes (Fig. 3c). These results together indicated the accuracy and generality of *EpiCas-DL* in sgRNA activity prediction of gene silencing for epigenome editing tools.

### 3.3 The ability of *EpiCas-DL* to predict sgRNA activity for CRISPRa

**Fig. 4:**
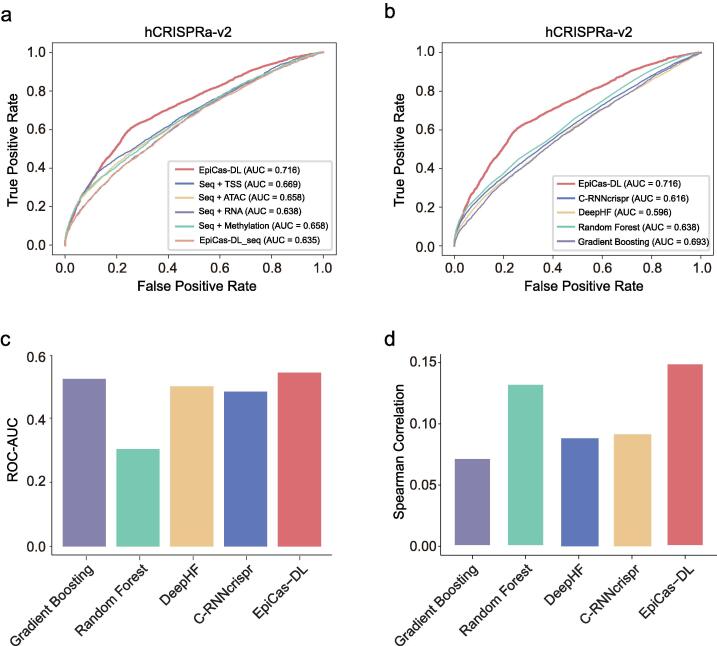
Implementation details of *EpiCas-DL* on CRISPRa dataset. (a) Performance comparison of *EpiCas-DL* models with different epigenetic features in the hCRISPRa-v2 dataset using classification schema. “seq” represents sequence. (b) Performance comparison of different models on the sgRNA activity prediction in the hCRISPRa-v2 dataset using classification schema. (c)-(d) Comparison of the prediction performance of different models on the sgRNA activity in gene activation in the CRISPRa_activityscore dataset under the classification (c) or regression (d) schema.

We then applied the same approach to building an *EpiCas-DL* model for sgRNA activity prediction in gene activation using the hCRISPRa-v2 dataset containing 198,756 sgRNAs across 18,496 genes (Table S3). Unlike the model built for sgRNA activity prediction in gene silencing cohorts, the *EpiCas-DL* model with 23mer length of input sequences showed the best performance for gene activation efficiency prediction (Fig. S4a). Similar to the results in gene silencing datasets, the model integrating sequence and all epigenetic features showed the highest prediction accuracy for both classification and regression models (Fig. 4a and S4b). We next compared the performance of the *EpiCas-DL* model in predicting the sgRNA activities of the hCRISPRa-v2 dataset (training) and an independent dataset CRISPRa_activityscore (testing). The loss function converged fast during training and validation process (Fig. S4c). In comparison with other machine learning- or deep learning-based models, *EpiCas-DL* achieved much higher ROC-AUC in both the training dataset and independent validation set (Fig. 4b and c). Even though the *EpiCas-DL* regression model showed similar performance to the *Random Forest* algorithm in the training set (Fig. S4d), *EpiCas-DL* outperformed all the other models evaluated by Spearman correlation coefficients in the validation cohort (Fig. 4d).

### 3.4 Importance features associated with epigenome editing outcome

**Fig. 5:**
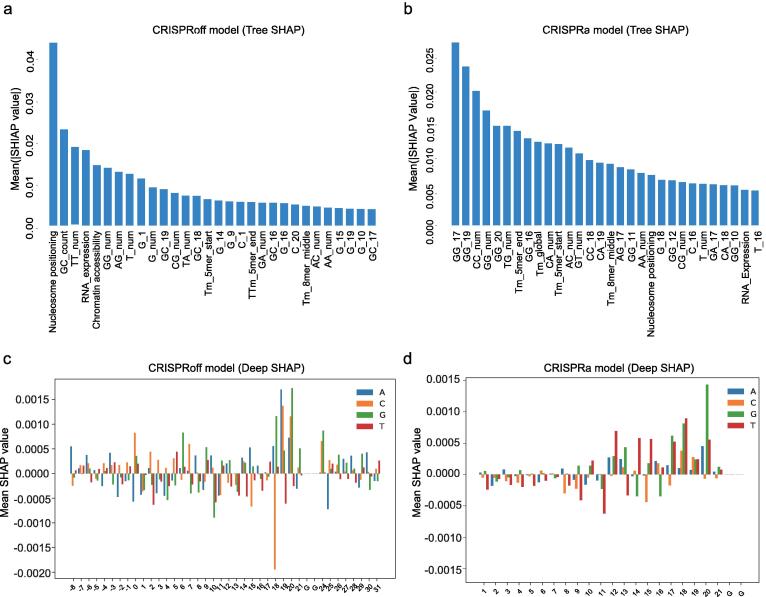
Feature importance associated with sgRNA activity in gene silencing or activation. (a)-(b) Top 30 features identified by Tree SHAP contributing to sgRNA activities in the CRISPRoff (a) or CRISPRa (b) dataset. For sequence features, the nucleotides together with their positions were shown on the x-axis, e.g., GG_17 represents GG dimer at position 17 (PAM as 21–23). Tm, melting temperature. Mean (|SHAP value|) represents the average impact on the model output magnitude. (c)-(d) Position-specific sequence feature importance is resolved by Mean SHAP values for the EpiCas-DL regression model in the CRISPRoff (c) or CRISPRa (d) dataset by DeepSHAP. A positive value indicates favored feature, and a negative value indicates disfavored feature.

We next explored which features contributed most to the sgRNA activity of gene silencing or activation in our *EpiCas-DL* models. We applied the SHapley Additive exPlanations (SHAP) method to generate a feature saliency map for sgRNA on-target efficiency [27]. The global SHAP values using Tree SHAP suggested that nucleosome positioning estimated by distance to TSS, RNA expression levels, and chromatin accessibility estimated by ATAC-seq ranked as the top five favorable features for efficient editing of CRISPRoff (Fig. 5a and Table S4). These results indicated that the accessibility of sgRNA binding to the target site plays an important role in the CRISPR-mediated epigenome editing system, in line with the characteristics of CRISPR-Cas editing system reported before [18], [19]. By contrast, the top-ranked characteristics favored by CRISPRa were sequence features instead of epigenetic features (Fig. 5b), which could be explained by the incremental effects of epigenetic characteristics in the CRISPRa modeling (Fig. 4). Consistent with previous studies [17], we found Tm values, GC counts and GC di-nucleotides also ranked as top important features associated with gene silencing by CRISPRoff (Fig. 5a and Table S4). Among the sequence features contributing to gene silencing and activation, we found that most of the position-related features were close to the PAM region (Fig. 5a, b), consistent with a previous study showing that sequences close to PAM tended to be more predictive of Cas9/gRNA complex activity [17]. One possible explanation is that the PAM proximal sequence marks how fast a specific Cas9/sgRNA complex finds its target, and thus influences the activity of Cas9 [17]. Another explanation could be the thermodynamic properties defined by gRNA-DNA hybridization free energy, and high binding free energies determined by PAM proximal sequence contribute to high editing efficiency [44], [45], [46].

Simultaneously, the contribution of each position-dependent nucleotide to sgRNA activity was calculated from the average of 1000 randomly selected sgRNAs in the training data using DeepSHAP [27], and nucleotide features influencing the predictive efficiency of the CRISPRoff tool were identified (Fig. 5c). For nucleotides adjacent to PAM sequence (positions 20 and 24), guanine is favored for highly efficient sgRNA (Fig. 5c). Similarly, guanine was also favored at position 20 of the guide for CRISPRa model (Fig. 5d). These observations were consistent with previous findings of CRISPR/Cas9 applications in eukaryotic cells [47], [48], [49], but disagreed with those found in bacteria [50], [51]. The favor of G proximal to the PAM for efficient gene silencing or activation by dCas9 associated epigenome editing systems was in line with the strong binding free energies for G proximal to the PAM [44], [46]. Besides, a preference for adenine was observed at positions from the middle to PAM of sgRNAs, and T and C were disfavored (Fig. 5c). The bias against thymine might be explained by the limited sgRNA expression at uracil-rich regions by RNA polymerase III termination [8], [47], [52]. Despite the similarity in some sequence features, CRISPRa model differed from CRISPRoff for per-base nucleotide preference. For examples, cytosine is disfavored at positions 19 and 20 of CRISPRa model, while T is disfavored for CRISPRoff (Fig. 5c, d). In addition, T was favored for CRISPRa at positions 12, 14 and 15, but was disfavored by CRISPRoff at these positions (Fig. 5c, d), suggesting that the determinants of CRISPRa and CRISPRoff efficiency were associated with fused proteins in addition to the preferences of dCas9. These results demonstrated that epigenetic and sequence features at specific positions are preferred for optimized sgRNA design in gene silencing or activation assay.

## 4 Discussion

Accurately predicting the targeting efficiency of sgRNAs is a major goal of genetic research in CRISPR applications. A large number of algorithms have been designed for cleavage dependent CRISPR/Cas gene editing systems rather than CRISPR-mediated epigenome editing. Therefore, here we developed a convolutional neural network-based model, *EpiCas-DL*, for predicting the sgRNA activities of CRISPRoff, CRISPRi, and CRISPRa systems. The convolutional layers of *EpiCas-DL* are composed of multiple-size filters to obtain sufficient information from the contextual sequences. Importantly, *EpiCas-DL* takes into consideration the epigenetic features that are important for gene silencing or activation effects mediated by epigenome editing tools, and the performance of *EpiCas-DL* also surpassed the other state-of-art algorithms. In addition, *EpiCas-DL* identifies specific features that contributed to high efficiency of sgRNAs and thus optimizes the on-target design of CRISPR-mediated epigenome editing systems in future application.

Unlike the typical CRISPR-Cas editing systems, the CRISPR-mediated epigenome editing tools regulated the expression of targeted genes by DNA methylation and histone modification factors [12], [53]. This process is prone to be affected by cellular characteristics like chromatin accessibility, DNA methylation status, endogenous gene expression levels, and nucleosome occupancy at the target site in addition to the sequence context. The sequence features associated with highly efficient epigenome editing were generally consistent with previously identified signatures of CRISPR/Cas9 [46], [47], [48], [49], Cpf1 [19] and dCas9-mediated gene silencing [47] in eukaryotic cells. While, we observed a strong favor to guanine immediately 3′ of the PAM, against with a previous study showing disfavor to guanine at this position [47], [50], [51]. Given that the previous study considered only sequence features for gene silencing efficiency prediction, the difference might result from the influence of epigenetic factors that were incorporated into our *EpiCas-DL* model. Indeed, our results demonstrated the importance of these epigenetic features in affecting the editing efficiency of CRISPRoff, CRISPRi, and CRISPRa systems. While, some of these features like nucleosome occupancy are not unique to CRISPR-mediated epigenome editing tools, but are expected to be generally important for most Cas9-mediated applications by affecting the binding of Cas9 [23]. The ability of *EpiCas-DL* to predict sgRNA activity for gene silencing in different cell lines and endogenous targeted genes from independent datasets indicated the reliability and generality of our model on a broader scale and application possibility in biological experiments.

Despite the robust performance of *EpiCas-DL*, several future improvements are expected, (1) Although the structure of *EpiCas-DL* is based on CNN, other more complex deep learning frameworks, such as recurrent neural networks (RNNs), generative adversarial networks (GANs), and reinforcement learning (RL), are waiting to be explored in improving our current prediction performance [26]. (2) Epigenomics is a complex system influenced by many factors. In this study, we only considered nucleosome positioning, chromatin accessibility, DNA methylation, and RNA expression as the main features that affect epigenome editing. The performance of *EpiCas-DL* models was also limited by the incomplete information for these epigenetic features. Future directions considering more complete epigenetic features with few missing values are expected to improve the prediction performance. (3) The amount of available CRISPRoff screening datasets were relatively small and mainly conducted in HEK293T and K562 cell lines, which may increase the overfitting risk in the training process and thus limit the generalization ability. More diverse data sources including various cell types and organisms could possibly improve the performance of *EpiCas-DL* for efficiency prediction in gene silencing or activation assays like a recent study for CRISPR/Cas9 sgRNA activity prediction [49]. (4) The differences between CRISPRoff and CRISPRi systems might affect the prediction performance of *EpiCas-DL* in the testing datasets. (5) The gene silencing and activation screening datasets may contain noises in the process of measuring the sgRNA knock-down or knock-up efficacy, and these underlying confounding factors need to be further explored.

The focus of our current research is to demonstrate *EpiCas-DL* as the first epigenetic editing predictive model for CRISPR-mediated epigenome editing systems. We further envision replacing or adding the epigenetic information of the guide or multiple sgRNA combinations in future models. Going forward, it could be fruitful to increase the research of epigenomes by designing active sgRNA for CRISPR-associated epigenome editing systems.

## 5 Conclusion

In this study, we introduced *EpiCas-DL*, a deep-learning based model developed to optimize sgRNA design for CRISPR-mediated targeted gene silencing or activation activity. We demonstrated the sensitivity and specificity of *EpiCas-DL* in various gene silencing or activation screening datasets, and *EpiCas-DL* outperformed other available *in silico* methods. Meanwhile, epigenetic and sequence features that contribute to the gene silencing and activation activities were identified. To facilitate the utilization of *EpiCas-DL* to a broad biomedical community, we also provide an interactive website for rapid and convenient exploration at http://www.sunlab.fun:3838/EpiCas-DL.

## Declarations

### 6 Ethics approval and consent to participate

Not applicable.

### 7 Consent for publication

Not applicable.

### 8 Availability of data and materials

*EpiCas-DL* is implemented as Python packages, and it is freely available under the MIT license on https://github.com/yangqianq/EpiCas-DL. The sgRNA related datasets analyzed in this study are shared in the supplementary files with their sources [12], [20], [36]. The RNA expression, ATAC, methylation feature datasets under following accession numbers: GSE152177 [28], GSM720355 [29], GSE114071 [30], GSM1589167 [31], and ATACdb Sample_0101. The TSS feature dataset used in this study is listed in Table S2 from published article of Max A Horlbeck et al [20]. The EpiCas-DL website is freely accessible at https://www.sunlab.fun:3838/EpiCas-DL.

## 9 Competing interests

The authors disclose a patent application relating to aspects of this work.

## Funding

This study was supported by the 10.13039/501100001809Young Scientists Fund of the National Natural Science Foundation of China (Grant No. 32100487), and Shanghai Municipal Science and Technology Major Project (Grant No. 2018SHZDZX05).

## CRediT authorship contribution statement

**Qianqian Yang:** Data curation, Formal analysis, Investigation, Methodology, Software, Writing – original draft. **Leilei Wu:** Formal analysis, Methodology. **Juan Meng:** Software. **Lei Ma:** Methodology. **Erwei Zuo:** Methodology, Writing – review & editing. **Yidi Sun:** Conceptualization, Funding acquisition, Investigation, Methodology, Supervision, Writing – review & editing.

## Declaration of Competing Interest

The authors declare that they have no known competing financial interests or personal relationships that could have appeared to influence the work reported in this paper.
