# Supplementary figures and images for: EpiCas-DL: Predicting sgRNA activity for CRISPR-mediated epigenome editing by deep learning
Source: Comput Struct Biotechnol J. 2022 Nov 19;21:202–11. doi: 10.1016/j.csbj.2022.11.034 (PMC9763632; doi:10.1016/j.csbj.2022.11.034)

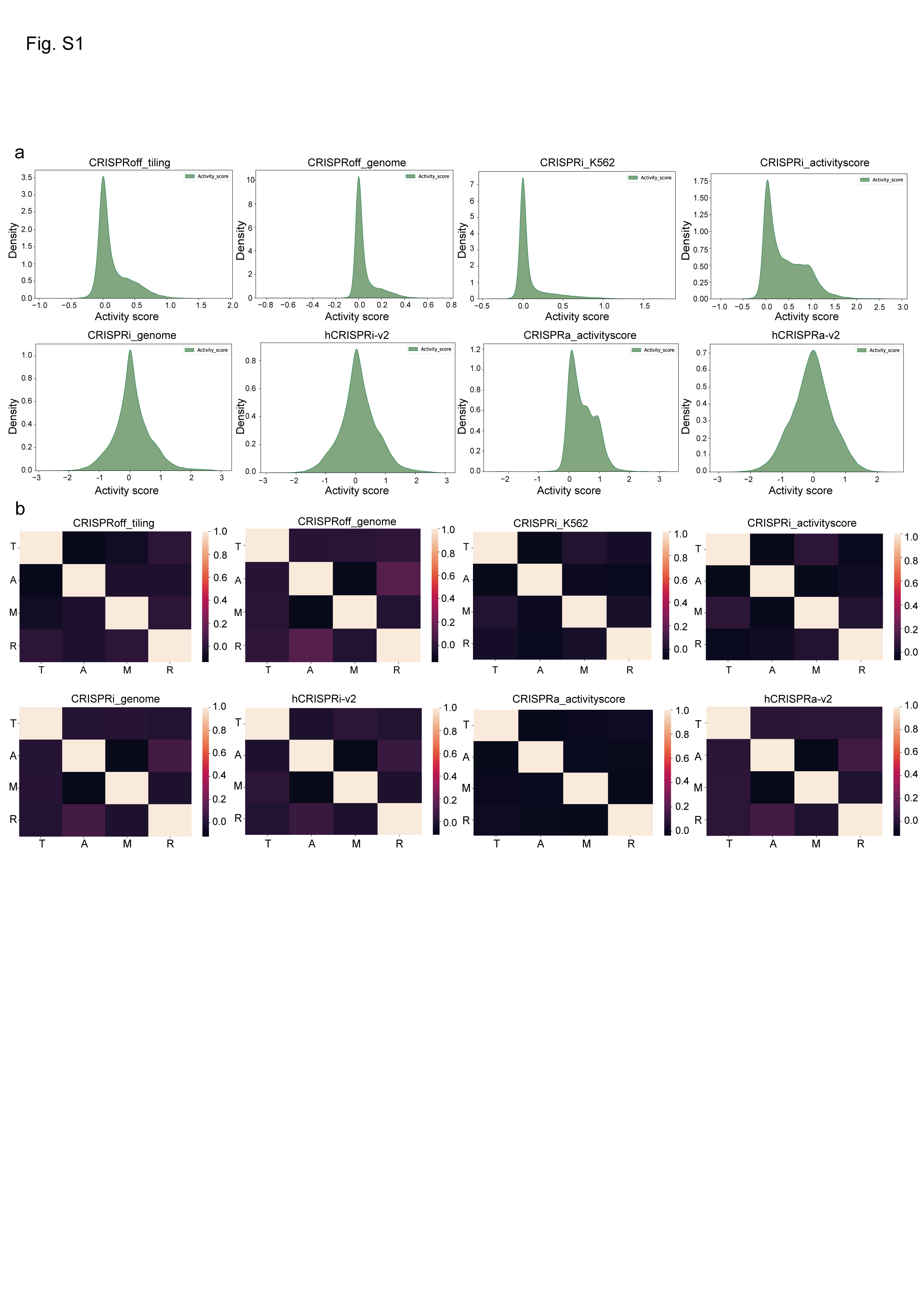

Supplement: Supplementary data 5 [file mmc5.zip › FigureS1.png]

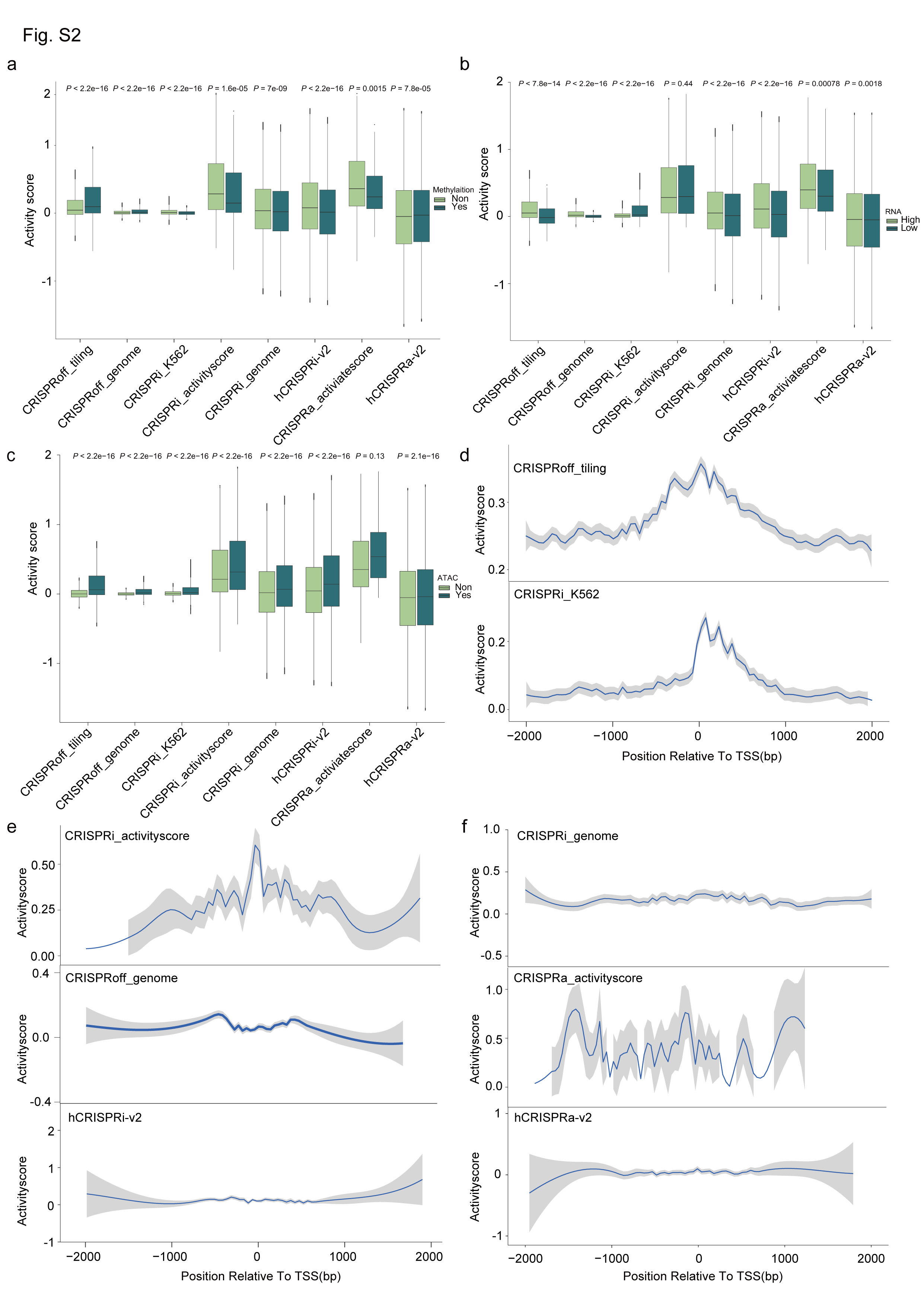

Supplement: Supplementary data 5 [file mmc5.zip › FigureS2.png]

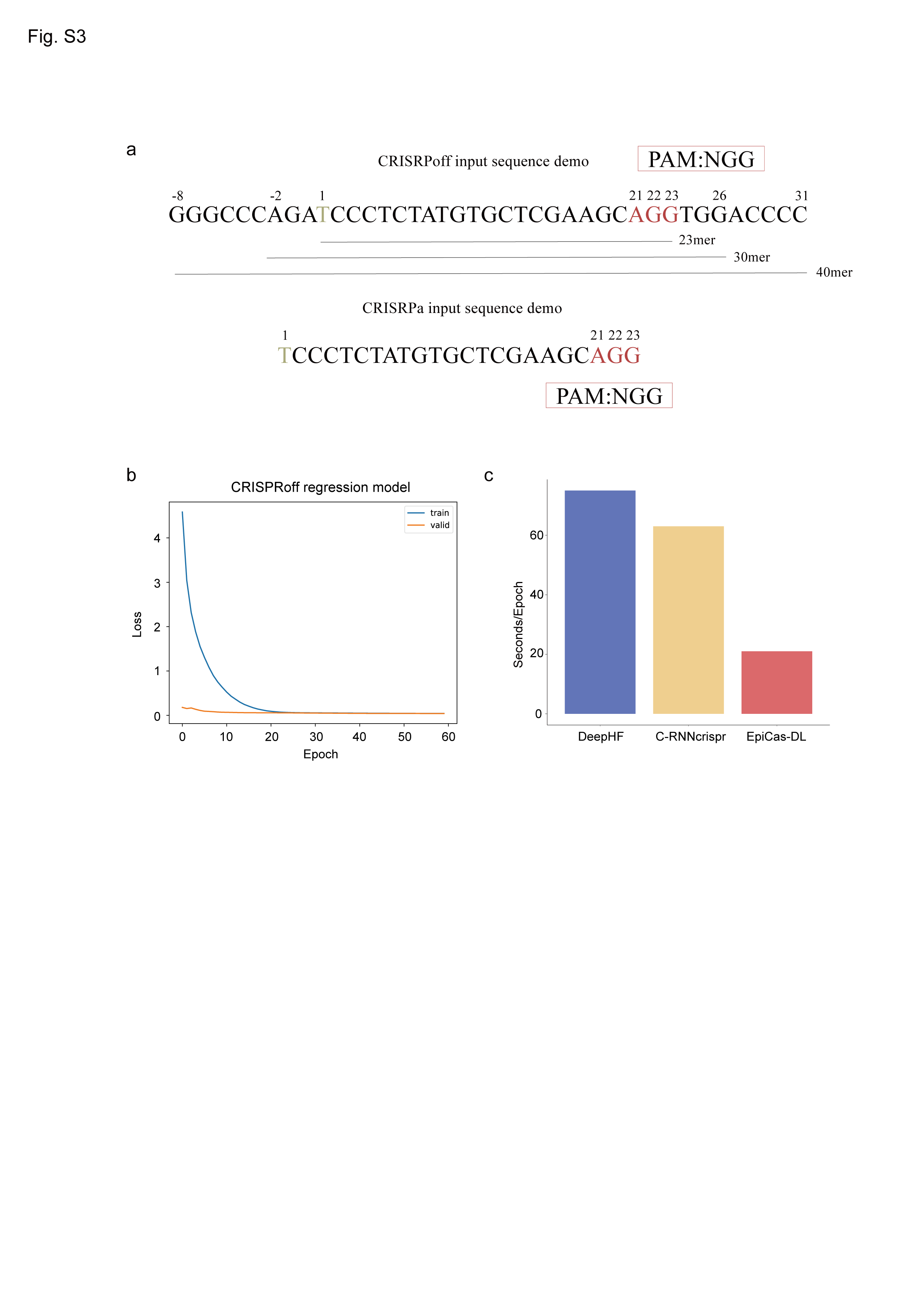

Supplement: Supplementary data 5 [file mmc5.zip › FigureS3.png]

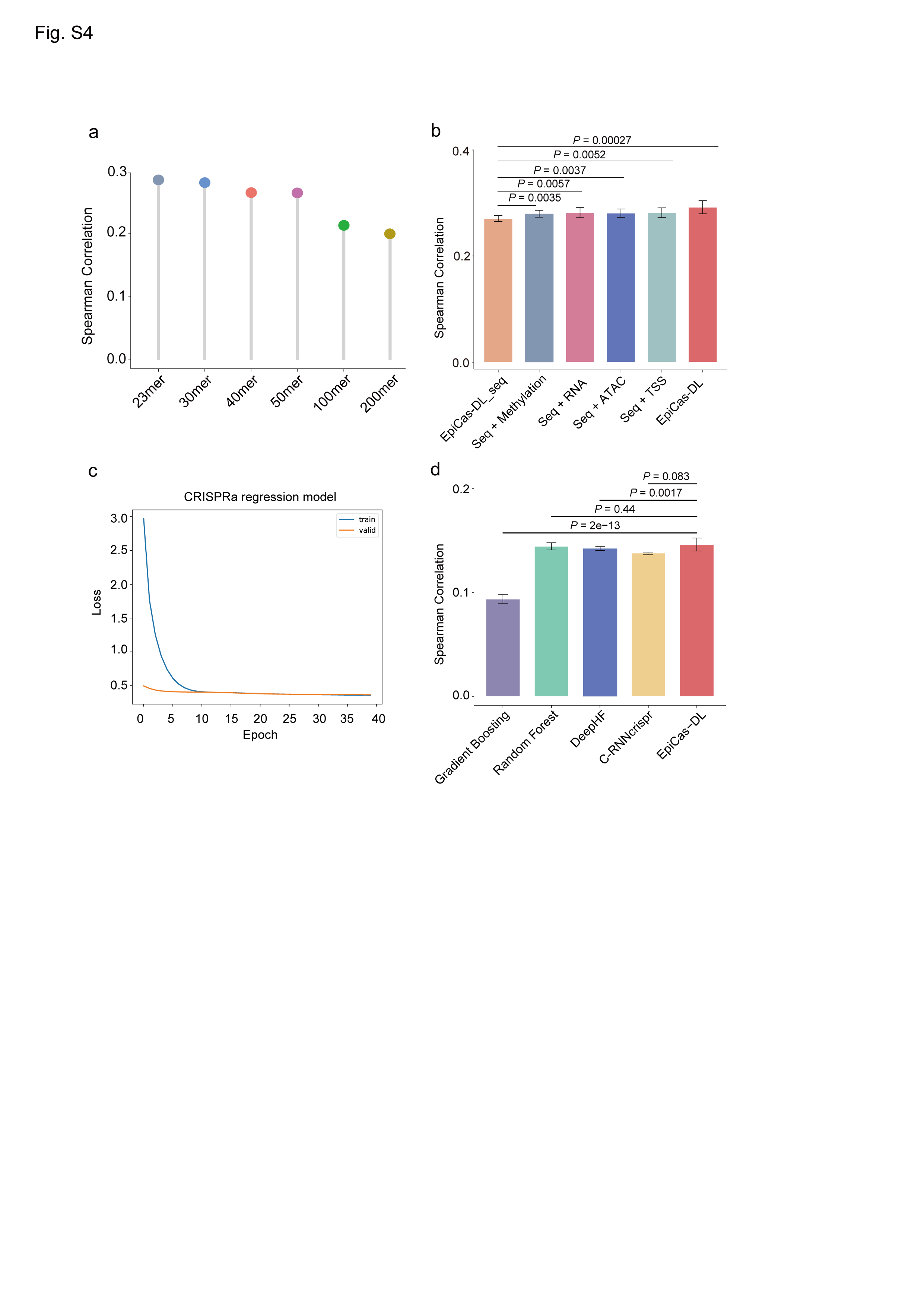

Supplement: Supplementary data 5 [file mmc5.zip › FigureS4.png]
